# A Study on the Association Between Polymorphisms in the Cytochrome P450 Family 17 Subfamily A Member 1 Gene Region and Type 2 Diabetes Mellitus in Han Chinese

**DOI:** 10.3389/fendo.2018.00323

**Published:** 2018-06-11

**Authors:** Long Wang, Yu-Ming Niu, Shi-Shi Wu, Chao Zhang, Li Zhou, Hong-Xia Zuo, Peng Wang

**Affiliations:** ^1^Department of Evidence-Based Medicine and Clinical Research, Taihe Hospital, Hubei University of Medicine, Shiyan, China; ^2^Department of Histology and Embryology, School of Basic Medical Sciences, Hubei University of Medicine, Shiyan, China; ^3^Research Center for Medicine and Social Development, School of Public Health and Management, Chongqing Medical University, Chongqing, China; ^4^Department of Clinical Laboratory, Taihe Hospital, Hubei University of Medicine, Shiyan, China

**Keywords:** cytochrome P450 family 17 subfamily A member 1, polymorphism, type 2 diabetes, susceptibility, steroid hormone

## Abstract

**Background:**

Cytochrome P450 family 17 subfamily A member 1 (CYP17A1) gene encodes a key enzyme in the synthesis and metabolism of steroid hormones and has been associated with various factors, such as hypertension, insulin resistance, and polycystic ovary syndrome. However, whether the gene was associated with type 2 diabetes mellitus (T2DM) has not been reported yet. Therefore, we sought to investigate whether CYP17A1 was associated with T2DM and related traits among Han Chinese.

**Methods:**

Three tagging single nucleotide polymorphisms (rs1004467, rs17115149, and rs12413409), in the CYP17A1 gene region were selected and genotyped in a case–control study that included 440 diabetes and 1,320 control subjects. Effects of genetic loci were studied using univariate unconditional logistic regression and multivariate logistic regression analysis adjusted for age, sex, family history, body mass index, smoking, and drinking. Bioinformatics analysis was also conducted using the GEO DataSets and PROMO database to gain hints of possible mechanism.

**Results:**

Rs17115149 and rs12413409 polymorphisms were significantly associated with the risk of T2DM, even after adjusting for age, sex, family history, body mass index, smoking, and drinking. In stratified analyses, rs1004467 and rs12413409 showed significant association with T2DM in the older age group (≥65 years) and, in the case of rs12413409, the risk of T2DM was significant in men but not in women. Rs17115149 had significant association with T2DM in the hypertension subgroup, and rs12413409 in the non-hypertension subgroup. Moreover, rs12413409 showed significant association with plasma glucose levels in the recessive model (*P* = 0.020) among subjects not taking hypoglycemic measures. Bioinformatics analysis revealed significantly higher CYP17A1 gene expression in T2DM patients compared to healthy controls. Finally, the mutant T allele of the rs17115149 polymorphism allowed binding to the RBP-Jkappa transcription factor.

**Conclusion:**

This is the first report to identify that variants rs1004467, rs17115149, and rs12413409 of CYP17A1, are related to plasma glucose levels and T2DM among Han Chinese. Our results suggest that CYP17A1 might constitute a risk gene for progression to T2DM.

## Introduction

Type 2 diabetes (T2DM) is a complex multifactorial disorder caused by various susceptibility genes and a variety of environmental determinants, and is one of the main challenges of modern health (1). There is convincing evidence that genetic factors contribute strongly to an individual’s risk of developing T2DM (2). Several large-scale association studies have reported numerous common, rare, and functional variants of T2DM (3, 4). To date, more than 100 susceptibility loci have been identified to influence the risk for T2DM, and recent studies have argued that many additional risk loci remain to be determined (5).

The cytochrome P450 family 17 subfamily A member 1 (CYP17A1) gene, located on chromosome 10q24.3, consists of eight exons and seven introns and is expressed mainly in the adrenal glands and gonads (6, 7). In humans, CYP17A1 encodes the P450c17 protein, a key enzyme in the steroidogenic pathway. It can catalyze two distinct types of substrate oxidation (8, 9): 17alpha-hydroxylation of steroids and the 17,20-lyase reaction, which are essential for corticoid biosynthesis and sex steroid precursors generation, respectively (10). Some studies show that deficient expression of P450c17 can impair androgen, estrogen, and cortisol hormone synthesis, while producing excessive amounts of mineralocorticoid, and may cause hypertension, hypokalemia, pseudohermaphroditism, and delayed sexual maturation (11, 12).

Genetic association studies have revealed that the CYP17A1 gene plays an important role in various pathological conditions, such as visceral and subcutaneous fat accumulation (13), coronary artery disease (CAD) (14), hypertension (12), prostate cancer (15), insulin resistance, and polycystic ovary syndrome (16), which are often related to T2DM. Moreover, considering that corticoids are also associated with glycometabolism, CYP17A1 is likely to be involved in T2DM. However, no studies have investigated the relationship between CYP17A1 and T2DM in the Han Chinese population. Therefore, here, we aimed to assess the association between CYP17A1 polymorphisms and T2DM among Han Chinese.

## Materials and Methods

### Study Subjects

An age- (±5 years) and sex-frequency matched case–control study was conducted, which included 440 T2DM patients and 1,420 non-diabetic controls. Patients and controls were recruited from three hospitals in Chongqing city between October 2013 and July 2015. The three hospitals were the Second Affiliated Hospital of Chongqing Medical University, the Chongqing Zhongshan Hospital, and the Chongqing Hospital of Traditional Chinese Medicine. All type 2 diabetic patients included in the study had to meet the 1999 WHO criteria for diabetes (17): a fasting glucose level ≥7.0 mmol/L or a 2-h glucose level ≥11.1 mmol/L, treatment with insulin and/or oral hypoglycemic agents following a diagnosis of T2DM, and having been diagnosed after the age of 35 years. The non-diabetic controls resided in the same communities as the cases, and inclusion criteria were as follows: (1) >50 years of age, (2) a fasting glucose level <6.1 mmol/L or 2-h glucose level <7.8 mmol/L and no family history of T2D, (3) no past history of diabetes, and (4) no severe liver disease and/or kidney disease. All participants underwent a questionnaire-based interview aimed at collecting their family history, medical history, as well as information pertaining to medication, home environment, and lifestyle factors.

Subjects who had smoked >100 cigarettes or had drunk <3 times per week for >1 year in their lifetime were defined as smokers and drinkers, respectively. Notably, during the interview, some subjects whose systolic and diastolic blood pressure measured slightly >140 and >90 mmHg, respectively, but had never been diagnosed with hypertension, were classified as the uncertain hypertension group. This study was approved by the ethics committee of Chongqing Medical University and was performed in accordance with the guidelines of the Helsinki World Medical Association Declaration. After a full explanation of the study, all participants approved the study and written informed consents were obtained.

### Laboratory Testing

Blood samples were collected from all participants after overnight fasting (at least 12 h). Then, extensive anthropometric and biochemical traits related to glucose metabolism were measured using standard laboratory procedures in the clinical laboratories of the respective hospitals. Parameters included blood pressure, fasting blood glucose (FBG), total cholesterol, high-density lipoprotein cholesterol, low-density lipoprotein cholesterol, and triglyceride.

### Single Nucleotide Polymorphism (SNP) Selection

Three tagging SNPs, rs1004467, rs17115149, and rs12413409, were selected using the pairwise tagging method in Haploview 4.0, based on *R*^2^ < 0.8 and minor allele frequency >0.05 across the CYP17A1 gene region using 1,000 Chinese Han population genome data sets. The selected SNPs were the most frequently analyzed SNPs at this locus in the Chinese population.

### DNA Extraction and Genotyping

Human genomic DNA samples were extracted from peripheral blood (QIAamp DNA blood kit; QIAGEN, Hilden, Germany) according to the manufacturer’s instructions. A NanoDrop 2000 spectrophotometer (Thermo Scientific, DE, USA) was used to measure the concentration. The eluted and qualified DNA samples were stored at −80°C prior to further use.

Genotyping was performed using a MassARRAY time-of-flight mass spectrometer (Sequenom, San Diego, CA, USA), as described in our previous study (18). Success rates for rs1004467, rs17115149, and rs12413409 were 98.8, 100.0, and 98.8%, respectively.

### Bioinformatics Analysis of CYP17A1 Gene Expression and rs17115149 Polymorphism Function

We conducted a bioinformatics analysis to further explore the possible mechanisms of the CYP17A1 gene and rs17115149 variant. We used the GEO DataSets (https://www.ncbi.nlm.nih.gov/gds/) to explore CYP17A1 gene expression in type 2 diabetic patients compared with non-diabetics, and the PROMO database (http://alggen.lsi.upc.es/cgi-bin/promo_v3/promo/promoinit.cgi?dirDB=TF_8.3) to inspect whether the rs17115149 polymorphism included any transcription factor binding sites (TFBS). If so, a variant in this allele could cause gain/loss of binding to TFBS in humans.

### Statistical Analysis

For baseline characteristics, continuous variables were reported as mean ± SD, and categorical variables were reported as frequencies in percentages. Normal distribution of data was analyzed using the Kolmogorov–Smirnov normality test. Student’s *t*-test was used to compare the data with a normal distribution, and data with unequal variance and/or without a normal distribution were assessed using the Mann–Whitney rank sum test. The chi-square test and Hardy–Weinberg equilibrium (HWE) analyses were used for categorical variables. Univariate unconditional logistic regression analysis was performed to compare the case and control groups by computing the odds ratios and their 95% confidence intervals (CIs). Adjusted logistic regression analysis was conducted after adjusting for age, sex, family history, body mass index, smoking, and drinking. Each model was composed of allele A versus allele B, with A being the major allele and B the minor allele. This generated the following models: dominant (AB + BB versus AA), recessive (BB versus AB + AA), codominant (BB versus AA and AB versus AA), overdominant (AA + BB versus AB), and addictive (AA versus AB versus BB). A haplotype analysis of rs1004467, rs17115149, and rs12413409 SNPs was performed using Phase 2.0 software (19). The association between FBG and variant genotypes were measured using the Mann–Whitney rank sum test. We used the Haploview 4.0 program to analyze pairwise linkage disequilibrium (LD) based on data extracted from 1,000 genomes. A *P*-value <0.05 was defined as significant. Statistical analyses were performed with SPSS software version 16.0 (SPSS Inc., Chicago, IL, USA).

## Results

### Characteristics of the Subjects and Variants

Table 1 lists the baseline characteristics of study participants. Among the 1,860 participants, 440 were type 2 diabetic patients and 1,420 were non-diabetic controls. The mean ages of the case and control groups were 70.04 and 66.32 years, respectively. No significant difference was observed in sex distribution between diabetic and control groups (*P* = 0.660). Diabetic cases had higher FBG and systolic blood pressure levels and higher rates of hypertension, CAD, and hyperlipidemia compared to non-diabetic controls.

**Table 1:** Baseline characteristics of type 2 diabetes mellitus cases and non-diabetic controls.

Variables	Cases (*N* = 440)	Controls (*N* = 1,320)	*P*-value
Sex, m/f, (%)	224/216 (50.9/49.1)	656/664 (49.7/50.3)	0.660
Age, mean ± SD	70.04 ± 10.06	66.32 ± 12.22	<0.001
Body mass index (kg/m^2^)	23.47 ± 3.13	23.28 ± 3.10	0.444
Smoking, no/yes, (%)	336/104 (76.4/23.6)	1,059/261 (80.2/19.8)	0.083
Drinking, no/yes, (%)	376/64 (85.5/14.5)	1,132/188 (85.8/14.2)	0.875
Fasting glucose (mmol/L)	9.21 ± 3.89	5.44 ± 1.00	<0.001
Total cholesterol (mmol/L)	1.80 ± 0.40	1.79 ± 0.41	0.774
Triglyceride (mmol/L)	1.92 ± 0.28	1.93 ± 0.28	0.710
High-density lipid (mmol/L)	1.31 ± 0.47	1.37 ± 0.78	0.545
Low-density lipid (mmol/L)	1.85 ± 0.36	1.93 ± 0.25	0.109
Systolic blood pressure (mmHg)	137.33 ± 20.43	132.83 ± 18.31	<0.001
Diastolic blood pressure (mmHg)	77.80 ± 12.26	78.32 ± 11.48	0.432
Hypertension, no/yes/uncertainty, (%)	94/333/13 (21.4/75.7/2.9)	506/750/64 (38.3/56.9/4.8)	<0.001
Coronary heart disease, no/yes, (%)	141/299 (32.0/68.0)	699/621 (53.0/47.0)	<0.001
Hyperlipidemia, no/yes, (%)	286/154 (65.0/35.0)	930/390 (70.5/29.5)	0.032

The characteristics of the three selected SNPs are shown in Table S1 in Supplementary Material, and no apparent deviations in genotype distributions were observed based on HWE analysis for all SNPs in the control group (*P* > 0.05). The LD pattern of the three SNPs is shown in Figure S1 in Supplementary Material among the 1,000 genome of Chinese Han population, the diabetic group and control group. Rs1004467 and rs12413409 SNPs displayed moderate LD, whereas rs1004467 and rs17115149 exhibited no LD, even though their distance was relatively close.

### Genotype Analysis of CYP17A1 Gene Polymorphisms and T2DM

As presented in Table 2, among the selected three SNPs, univariate analysis and adjusted logistic regression analysis both indicated that rs17115149 and rs12413409 were significantly associated with T2DM. The risk genotype of rs17115149 was the GT variant (OR = 1.373; 95% CI, 1.020–1.849); moreover, the T allele appeared to be a risk allele when compared to the C allele (OR = 1.345; 95% CI, 1.029–1.760). After adjusting for multiple risk factors, rs17115149 was associated with T2DM in the dominant, overdominant, and addictive models. As for rs13413409, the AA genotype was found to pose a greater risk than the GG genotype (OR = 1.682; 95% CI, 1.122–2.522), and the association between rs12413409 and T2DM remained significant in the recessive model in multivariate analyses.

**Table 2:** Association of three genotyped single nucleotide polymorphisms with type 2 diabetes mellitus in the Han Chinese population.

Genotypes	Cases *n*(%)	Controls *n*(%)	Crude models		Adjusted models	
				
			OR (95% CI)	*P*-value	OR^a^ (95% CI)	*P*^a^-value
Rs1004467 T>C						
Codominant						
TT	188 (43.3)	578 (44.3)	1.00	–	1.00	–
TC	189 (43.5)	593 (45.5)	0.980 (0.777–1.236)	0.864	1.001 (0.791–1.265)	0.996
CC	57 (13.1)	133 (10.2)	1.318 (0.927–1.872)	0.124	1.400 (0.979–2.000)	0.065
Dominant	–	–	1.042 (0.837–1.297)	0.714	1.071 (0.858–1.337)	0.543
Recessive	–	–	1.331 (0.956–1.854)	0.091	1.399 (0.999–1.960)	0.051
Overdominant	–	–	1.081 (0.869–1.346)	0.485	1.071 (0.859–1.336)	0.542
Addictive	–	–	1.093 (0.929–1.286)	0.284	1.123 (0.952–1.324)	0.170
Allele	–	–	1.092 (0.929–1.284)	0.286	–	–

**Rs17115149 G>T**						
**Codominant**						
GG	361 (82.0)	1,139 (86.3)	1.00	–	1.00	–
GT	74 (16.8)	170 (12.9)	1.373 (1.020–1.849)	0.037*	1.950 (1.307–2.909)	0.001*
TT	5 (1.1)	11 (0.8)	1.434 (0.495–4.155)	0.506	1.023 (0.972–1.076)	0.385
Dominant	–	–	1.377 (1.031–1.840)	0.030*	1.887 (1.275–2.791)	0.001*
Recessive	–	–	1.368 (0.473–3.959)	0.563	0.959 (0.197–4.664)	0.959
Overdominant	–	–	0.731 (0.543–0.984)	0.039*	0.513 (0.344–0.766)	0.001*
Addictive	–	–	1.330 (1.022–1.731)	0.034*	1.684 (1.181–2.400)	0.004*
Allele	–	–	1.345 (1.029–1.760)	0.030*	–	–

**Rs12413409 G>A**						
**Codominant**						
GG	230 (53.2)	700 (53.6)	1.00	–	1.00	–
GA	160 (37.0)	530 (40.6)	0.919 (0.729–1.158)	0.473	0.927 (0.736–1.169)	0.524
AA	42 (9.7)	76 (5.8)	1.682 (1.122–2.522)	0.012*	1.738 (1.157–2.612)	0.008*
Dominant	–	–	1.014 (0.816–1.262)	0.897	1.027 (0.825–1.277)	0.815
Recessive	–	–	1.743 (1.176–2.584)	0.006*	1.795 (1.209–2.665)	0.004*
Overdominant	–	–	1.161 (0.928–1.453)	0.192	1.155 (0.923–1.446)	0.208
Addictive	–	–	1.116 (0.938–1.327)	0.216	1.130 (0.949–1.345)	0.169
Allele	–	–	1.114 (0.938–1.323)	0.219	–	–

***^a^**ORs and P values were calculated by logistic regression analysis with adjustment for age, sex, family history, body mass index, smoking, and drinking*.

*Indicates significant P-value.

*OR, odds ratio; CI, confidence interval*.

### Stratified Analyses Based on Age, Gender, and Hypertension

We then performed stratified analyses to explore the relationship between SNPs and conventional T2DM risk factors, including age, gender, and hypertension.

As shown in Table S2 in Supplementary Material, stratified analyses based on age and gender revealed that SNPs rs1004467 and rs12413409 were both significantly associated with T2DM in the older age group (≥65 years), whereas no significant associations were found in the other group (<65 years). Moreover, when the analysis was performed separately in men and women, significant associations of rs12413409 (codominant model and recessive model) and T2DM were observed in men, but not in women.

In the stratified analyses of hypertension, we found rs17115149 to be nominally associated with T2DM in the hypertension group, whereas rs12413409 was associated with T2DM in the non-hypertension group (Table 3).

**Table 3:** Stratified analysis of rs1004467, rs17115149, and rs12413409 polymorphisms on type 2 diabetes mellitus in the hypertension group.

Variable	Genotype models	Rs1004467		Rs17115149		Rs12413409	
				
		OR^a^ (95% CI)	*P*^a^-value	OR^a^ (95% CI)	*P*^a^-value	OR^a^ (95% CI)	*P*^a^-value
Hypertension							
No	Codominant	0.970 (0.736–1.278)	0.827	0.799 (0.385–1.657)	0.546	1.445 (0.877–2.381)	0.148
		1.056 (0.679–1.641)	0.810	0	0.999	2.705 (1.265–5.784)	0.010*
	Dominant	0.986 (0.759–1.281)	0.915	0.729 (0.353–1.505)	0.393	1.625 (1.013–2.606)	0.044
	Recessive	1.072 (0.704–1.631)	0.747	0	0.999	2.252 (1.104–4.591)	0.026*
	Overdominant	1.042 (0.801–1.355)	0.759	0.810 (0.390–1.680)	0.571	0.834 (0.524–1.329)	0.446
	Addictive	1.007 (0.827–1.226)	0.944	0.695 (0.351–1.374)	0.296	1.581 (1.112–2.248)	0.011*

Yes	Codominant	1.304 (0.783–2.173)	0.308	1.598 (1.127–2.266)	0.009*	0.864 (0.655–1.139)	0.299
		1.940 (0.933–4.034)	0.076	3.115 (0.825–11.751)	0.094	1.157 (0.663–2.020)	0.607
	Dominant	1.416 (0.872–2.299)	0.160	1.657 (1.179–2.330)	0.004*	0.898 (0.690–1.170)	0.427
	Recessive	1.677 (0.857–3.281)	0.131	0.346 (0.092–1.305)	0.117	1.229 (0.712–2.121)	0.460
	Overdominant	0.901 (0.565–1.436)	0.660	0.634 (0.447–0.899)	0.010*	1.174 (0.896–1.540)	0.245
	Addictive	1.371 (0.967–1.942)	0.076	1.631 (1.191–2.233)	0.002*	0.960 (0.772–1.193)	0.712

*^a^OR and P values were calculated by logistic regression analysis with adjustment for age, sex, family history, body mass index, smoking, and drinking*.

*Indicates significant P-value.

*OR, odds ratio; CI, confidence interval*.

### Haplotype Analysis of CYP17A1 Polymorphisms in T2DM

To determine whether the three SNPs in the CYP17A1 gene cluster accounted for any other associations with T2DM when tested together, a haplotype analysis for rs1004467, rs17115149, and rs12413409 in the T2DM and control groups was performed.

A total of eight haplotypes were found in both type 2 diabetic and control groups. The haplotypes with <1% frequency were excluded from further analysis. Finally, four haplotypes were compared between type 2 diabetic and control groups. As indicated in Table 4, compared with the haplotype TGG carriers, two other haplotype (TTG and CGG) carriers had significantly higher risk of diabetes (*P* < 0.05), with TTG displaying the strongest association with T2DM (OR = 1.70; 95% CI, 1.27–2.28; *P* = 3.09 × 10^−4^). However, no difference was found between CGA and TGG haplotypes in type 2 diabetic and control groups.

**Table 4:** Associations between three-site haplotypes in the cytochrome P450 family 17 subfamily A member 1 gene region and type 2 diabetes mellitus risk in Han Chinese participants.

Haplotype^a^	Case, n(%)	Control, n(%)	Odds ratio (95% CI)	*P*
TGG	469 (54.53)	1,566 (60.56)	1.00	
CGA	228 (26.51)	653 (25.25)	1.17 (0.97–1.40)	0.100
TTG	78 (9.07)	153 (5.92)	1.70 (1.27–2.28)	3.09 × 10^−4^
CGG	69 (8.02)	159 (6.15)	1.45 (1.07–1.96)	0.015

*^a^The sequence of single nucleotide polymorphisms is rs1004467, rs17115149, and rs12413409*.

### Associations Between CYP17A1 Polymorphisms and FBG Levels

**Figure 1:**
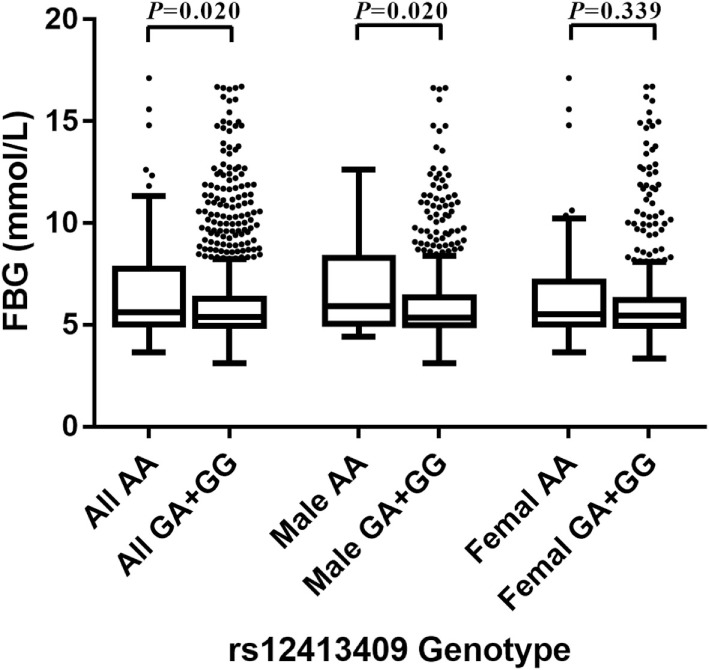
Box-whisker plot of fasting blood glucose (FBG) levels in the non-oral hypoglycemic agents and/or insulin takers study subgroup (*N* = 1,459), stratified by rs12413409 genotype and gender. The plot shows the median within the interquartile range box, with whiskers extending to the 5th and 95th percentiles; data points beyond the whiskers are displayed as dots. Groups were compared by the Mann–Whitney nonparametric test.

Among non-oral hypoglycemic agent and/or insulin takers, who included controls and T2DM patients not taking hypoglycemic measures, individuals with AA homozygous genotypes at rs12413409 exhibited significantly higher FBG levels than those with GA and GG carriers (*P* = 0.020). Furthermore, we found that the carriers of genotype AA had higher FBG levels compared to GA and GG genotype carriers in males (*P* = 0.020) but not in females (*P* = 0.339) (Figure 1).

Notably, rs1004467 and rs17115149 showed insignificant difference in FBG levels in different genotype carriers in all models.

### Bioinformatics Analysis of CYP17A1 Gene Expression and rs17115149 Polymorphism Function Prediction

**Figure 2:**
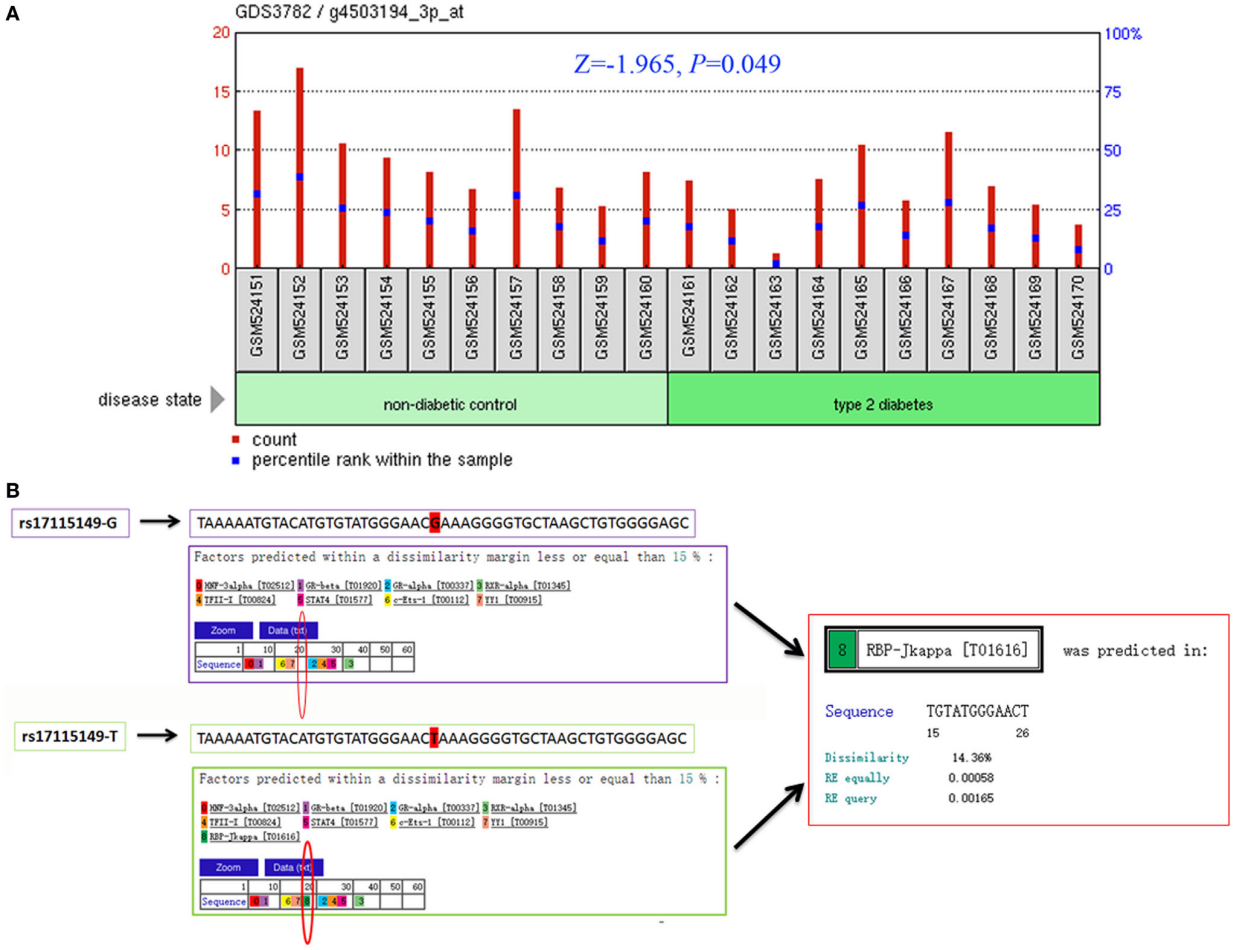
Bioinformatics analysis of the cytochrome P450 family 17 subfamily A member 1 (CYP17A1) gene and rs17115149 polymorphism. Analysis of CYP17A1 gene expression in type 2 diabetes mellitus patients compared with the healthy control group was performed using the high-throughput microarray gene expression database of GDS3782 data and Mann–Whitney *U* nonparametric test **(A)**. Bioinformatics analysis using data from the PROMO transcription factor binding site database showed that the mutant T allele at the rs17115149 polymorphism allowed binding to the RBP-Jkappa transcription factor **(B)**.

To determine CYP17A1 gene expression in T2DM patients compared with healthy controls, we analyzed the high-throughput microarray gene expression database of GDS3782 data from the GEO DataSets and found that the CYP17A1 gene expression was significantly higher in pancreatic beta-cells in T2DM patients compared to healthy controls (Figure 2A).

Considering that rs17115149 is located in the 5′ untranslated region of CYP17A1, which often harbors TFBS, the PROMO database was used to determine potential transcription factor binding to this SNP. Bioinformatics analysis showed that the mutant T allele at the rs17115149 polymorphism allowed binding to the RBP-Jkappa transcription factor (Figure 2B).

## Discussion

In the present study, we explored the potential relationship between CYP17A1 genetic polymorphisms and T2DM susceptibility. We report for the first time that SNPs rs17115149 and rs12413409 in the CYP17A1 gene cluster were strongly associated with T2DM risk in the Chinese Han population. Moreover, we observed that SNP rs17115149 was mainly responsible for the increased risk of T2DM among the hypertension group. Additionally, patients carrying rs12413409 AA genotypes had a higher FBG level and risk of T2DM among males. Consequently, our results indicate that CYP17A1 may be a candidate gene for T2DM susceptibility, and rs17115149 and rs12413409 polymorphisms may play an important role in the progression of T2DM.

Cytochrome P450 family 17 subfamily A member 1 encodes the P450c17 protein and plays a key part in the synthesis and metabolism of steroid hormones (10). Studies have shown that mutations in certain CYP17A1 sites could reduce the expression of P450c17, which may result in impaired androgen, estrogen, and cortisol hormone synthesis, while producing excessive mineralocorticoids, which may cause hypertension, hypokalemia, pseudohermaphroditism, and delayed sexual maturation (11, 12). Furthermore, several genetic association studies suggest that CYP17A1 plays an important role in different pathological conditions, such as visceral and subcutaneous fat accumulation (13), CAD (14), hypertension (12), prostate cancer (15), insulin resistance, and polycystic ovary syndrome (16). There is a strong epidemiologic, clinical, and phenotypic overlap between these conditions and T2DM. Furthermore, a large number of studies have shown that steroids, such as androgen (20), estrogen (21), cortisol (22), and mineralocorticoid (23) play important roles in the pathogenesis of diabetes. This evidence has raised the question of whether CYP17A1 contributed to the development of T2DM. So far, no obvious association between CYP17A1 gene polymorphisms and T2DM incidence has been observed. Wu et al. showed that serum P450c17 expression was lower in type 2 diabetic rats than in the normal control group (24). Ueshiba et al. showed that patients with T2DM had low 17,20-lyase and high 17α-hydroxylase activities (25). In the present study, we conducted bioinformatics analysis by analyzing the high-throughput microarray gene expression database of GDS3782 data, and found that CYP17A1 gene expression was higher in T2DM patients compared with the healthy control group. These findings reveal that CYP17A1 may contribute to the progression of T2DM. To further study the relationship between CYP17A1 genetic polymorphisms and T2DM, we choose three common genetic variants, rs1004467, rs17115149, and rs12413409 of CYP17A1, to explore their effects on risk of T2DM and related traits in the Han Chinese population.

Rs1004467, which is in the intron region of CYP17A1, has been associated with cardiovascular diseases. Specifically, two case–control studies have reported rs1004467 to be significantly associated with CAD in Chinese populations (14, 26). Furthermore, another case–control study found that rs1004467 in CYP17A1 was associated with arterial stiffness in 326 prediabetic and 743 diabetic subjects (27). In our study, we found that rs1004467 was associated with T2DM in the older age group (≥65 years). Our finding suggests that this variant might represent a genetic locus that plays a role in the development and progression of T2DM. Owing to the absence of additional studies on the rs1004467 polymorphism and T2DM, a large sample size association study and meta-analysis on rs1004467 with T2DM should be performed on Han Chinese in the future.

Rs17115149, a functional regulatory SNP, is located at −600 bp before the transcription site within the CpG islands of the CYP17A1 promoter. It has been associated with CYP17A1 RNA expression, and may represent a genetic risk factor for male infertility and testosterone levels (28). Furthermore, it is significantly associated with histologic aggressiveness and may be linked to development of prostate cancer (15). However, an association between rs17115149 and T2DM has never been reported, and its function remains unknown. In this study, we demonstrated that the polymorphism was associated with T2DM and could augment the risk of T2DM in the hypertension group, which suggests its importance in glucose metabolism. Using bioinformatics data from the PROMO transcription factor binding site database, we found that the mutant T allele at the rs17115149 locus allowed binding to the RBP-Jkappa transcription factor. RBP-Jkappa is a transcription-inhibiting factor of many target genes (29, 30) and has also been associated with the occurrence and development of diabetes mellitus (31, 32). Moreover, by analyzing the high-throughput microarray gene expression database of GDS3782 data, CYP17A1 gene expression was found to be higher in T2DM patients than in the healthy control group. Therefore, we hypothesize that the rs17115149 locus, located at the 5′ untranslated region of CYP17A1, may be associated with binding of RBP-Jkappa and, consequently, affect the expression of p450c17 and onset of T2DM. Rs17115149 may influence protein expression also *via* alternative splicing of mRNA.

The rs12413409 SNP is located in the CYP17A1-CNNM2-NT5C2 gene region on chromosome 10q24.32 and was associated with myocardial infarction (MI) in a Japanese population (33). It has also been associated with waist/hip ratio, heart rate, and MI in a Chinese population (34). Moreover, the association between this SNP and CAD has been confirmed within a Southern Han Chinese study (35). However, no research regarding the association between the SNP and T2DM has been reported so far. In the present study, significant association was found between rs12413409 and T2DM in the Han Chinese population, even after adjusting for age, sex, family history, body mass index, smoking, and drinking. Among non-oral hypoglycemic agent and/or insulin takers, rs12413409 polymorphism was significantly associated with plasma FBG levels, particularly in males. Further research is required to determine whether the association between CYP17A1 and T2DM is mediated through its effect on glucose metabolism.

Although we performed a rigorous case–control study to reveal a link and possible mechanisms between CYP17A1 polymorphisms and T2DM using meticulous multilevel statistics and bioinformatics analysis, there are also several limitations to our study. One of them is the lack of a functional and mechanistic investigation of rs17115149 and rs12413409. Therefore, future functional studies are warranted. In addition, during control group selection, we excluded individuals with a T2DM family history. Even though we adjusted the family history in the association analysis, such exclusion might induce a spurious association as it excludes genetically susceptible controls. Finally, as the association between CYP17A1 polymorphisms and T2DM has not been studied, the result of this study should be confirmed in a larger sample in the future.

In summary, the associations between CYP17A1 polymorphisms and T2DM and FBG levels described in this study have not been reported previously. To the best of our knowledge, this is the first report linking CYP17A1, which shows high affinity for steroid hormone metabolism and has been widely associated with cardiovascular disease, to glucose metabolism and the progression of T2DM.

## Conclusion

This study reveals that CYP17A1 rs17115149 and rs12413409 polymorphisms are associated with T2DM in the Han Chinese population. Furthermore, rs17115149 is associated with T2DM in the hypertension subgroup, and rs12413409 is associated with FBG levels. These observations suggest that CYP17A1 polymorphisms could be involved in glucose metabolism and increased risk of T2DM. The findings should be verified in further studies with larger and independent populations.

## Ethics Statement

This study was carried out in accordance with the recommendations of Medical Informed Consent Reference Guide with written informed consent from all subjects. All subjects gave written informed consent in accordance with the Declaration of Helsinki. The protocol was approved by the ethics committee of Chongqing Medical University.

## Author Contributions

Y-MN, S-SW, and LW conceived and designed the study, carried out the SNP genotyping and the statistical analysis of the genotype data, and drafted the manuscript. CZ and PW collected the data and contributed to the statistical analyses. S-SW and LZ participated in drafting the manuscript. H-XZ and LW revised the manuscript. All authors read and approved the final manuscript.

## Conflict of Interest Statement

The authors declare that the research was conducted in the absence of any commercial or financial relationships that could be construed as a potential conflict of interest.

## Supplementary Material

Figure S1 Linkage disequilibrium (LD) patterns between three genotyped single nucleotide polymorphism (SNPs). LD patterns between genotyped SNPs had shown among the 1,000 genome of Chinese Han population **(A)**, the diabetic group **(B)**, and control group **(C)**.
