# Supplementary figures and images for: A Study on the Association Between Polymorphisms in the Cytochrome P450 Family 17 Subfamily A Member 1 Gene Region and Type 2 Diabetes Mellitus in Han Chinese
Source: Front Endocrinol (Lausanne). 2018 Jun 11;9:323. doi: 10.3389/fendo.2018.00323 (PMC6004380; doi:10.3389/fendo.2018.00323)

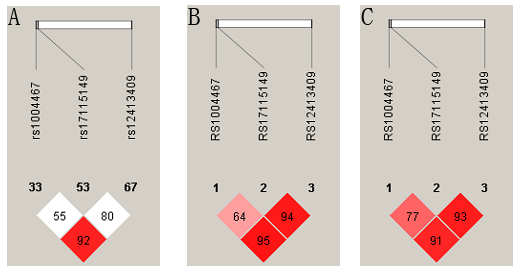

Supplement: Figure S1 — Linkage disequilibrium (LD) patterns between three genotyped single nucleotide polymorphism (SNPs). LD patterns between genotyped SNPs had shown among the 1,000 genome of Chinese Han population (A), the diabetic group (B), and control group (C). [file image_1.TIF]
