# Supplementary material for: A Study on the Association Between Polymorphisms in the Cytochrome P450 Family 17 Subfamily A Member 1 Gene Region and Type 2 Diabetes Mellitus in Han Chinese
Source: Front Endocrinol (Lausanne). 2018 Jun 11;9:323. doi: 10.3389/fendo.2018.00323 (PMC6004380; doi:10.3389/fendo.2018.00323)
Supplement: Supplementary file 2 [file table_1.DOC]

**Supplementary Table 1.** Characteristics of SNPs genotyped in CYP17A1 gene region

| SNP ID | Position | Nearby gene | Functional region | Allele A/B | HWE *P** |
| --- | --- | --- | --- | --- | --- |
| rs1004467 | Chr10:102834750 | CYP17A1 | intron | T/C | 0.289 |
| rs17115149 | Chr10:102837961 | CYP17A1 | 5'-UTR | G/T | 0.101 |
| rs12413409 | Chr10: 102959339 | CYP17A1, CNNM2, NT5C2 | intron | G/A | 0.062 |

Abbreviations: SNP, single nucleotide polymorphism; CHR, chromosome; HWE, Hardy–Weinberg equilibrium.

* The HWE *P* value was calculated in the control group.
