# Supplementary material for: A Study on the Association Between Polymorphisms in the Cytochrome P450 Family 17 Subfamily A Member 1 Gene Region and Type 2 Diabetes Mellitus in Han Chinese
Source: Front Endocrinol (Lausanne). 2018 Jun 11;9:323. doi: 10.3389/fendo.2018.00323 (PMC6004380; doi:10.3389/fendo.2018.00323)
Supplement: Supplementary file 3 [file table_2.DOCX]

**Supplementary table 2**. Stratified analysis of rs1004467, rs17115149, and rs12413409 polymorphisms on T2D in Han Chinese population

| Characteristics | Genotypes | Rs1004467 | | | | |  | Rs17115149 | | | | |  | Rs12413409 | | | | |
| --- | --- | --- | --- | --- | --- | --- | --- | --- | --- | --- | --- | --- | --- | --- | --- | --- | --- | --- |
|  |  | OR (95%CI) | *P* |  | OR^a^ (95%CI) | *P*^a^ |  | OR (95%CI) | *P* |  | OR^a^ (95%CI) | *P*^a^ |  | OR (95%CI) | *P* |  | OR^a^ (95%CI) | *P*^a^ |
| Age |  |  |  |  |  |  |  |  |  |  |  |  |  |  |  |  |  |  |
| <65 | Codominant | 0.772(0.514-1.160) | 0.213 | | 0.786(0.520-1.187) | 0.252 | | 1.405(0.837-2.358) | 0.198 | | 1.401(0.825-2.379) | 0.212 | | 0.812(0.542-1.216) | 0.312 | | 0.838(0.556-1.264) | 0.400 |
|  |  | 0.861(0.453-1.636) | 0.647 | | 0.996(0.516-1.922) | 0.990 | | 1.245(0.261-5.948) | 0.783 | | 1.172(0.240-5.724) | 0.844 | | 1.088(0.501-2.361) | 0.831 | | 1.158(0.526-2.553) | 0.716 |
|  | Dominant | 0.789(0.537-1.159) | 0.227 | | 0.822(0.556-1.215) | 0.325 | | 1.390(0.844-2.290) | 0.196 | | 1.379(0.828-2.294) | 0.217 | | 0.846(0.576-1.244) | 0.396 | | 0.877(0.592-1.298) | 0.510 |
|  | Recessive | 0.982(0.533-1.808) | 0.953 | | 1.125(0.602-2.103) | 0.712 | | 1.183(0.248-5.635) | 0.833 | | 1.115(0.229-5.434) | 0.892 | | 1.189(0.558-2.534) | 0.655 | | 1.250(0.578-2.703) | 0.571 |
|  | Overdominant | 1.256(0.853-1.849) | 0.248 | | 1.271(0.858-1.883) | 0.231 | | 0.714(0.426-1.198) | 0.203 | | 0.716(0.422-1.214) | 0.215 | | 1.243(0.837-1.846) | 0.280 | | 1.212(0.812-1.810) | 0.347 |
|  | Addictive | 0.870(0.648-1.167) | 0.352 | | 0.915(0.677-1.237) | 0.564 | | 1.306(0.844-2.019) | 0.230 | | 1.289(0.827-2.009) | 0.262 | | 0.920(0.669-1.263) | 0.605 | | 0.949(0.687-1.311) | 0.751 |
|  | Allele | 0.874(0.655-1.167) | 0.362 | |  |  | | 1.340(0.848-2.116) | 0.208 | |  |  | | 0.923(0.678-1.258) | 0.614 | |  |  |
| ≥65 | Codominant | 1.149(0.863-1.531) | 0.341 | | 1.170(0.877-1.561) | 0.286 | | 1.366(0.944-1.977) | 0.099 | | 1.350(0.932-1.956) | 0.112 | | 1.000(0.751-1.331) | 0.998 | | 1.014(0.761-1.351) | 0.924 |
|  |  | 1.692(1.096-2.613) | 0.018^⁎^ | | 1.701(1.100-2.630) | 0.017^⁎^ | | 2.416(0.484-12.051) | 0.282 | | 2.346(0.469-11.738) | 0.299 | | 2.029(1.235-3.333) | 0.005^⁎^ | | 2.038(1.237-3.356) | 0.005^⁎^ |
|  | Dominant | 1.246(0.951-1.633) | 0.111 | | 1.265(0.964-1.661) | 0.090 | | 1.399(0.974-2.010) | 0.070 | | 1.382(0.961-1.988) | 0.081 | | 1.129(0.863-1.478) | 0.377 | | 1.144(0.873-1.498) | 0.330 |
|  | Recessive | 1.579(1.048-2.380) | 0.029^⁎^ | | 1.573(1.043-2.373) | 0.031^⁎^ | | 2.307(0.463-11.496) | 0.307 | | 2.240(0.448-11.196) | 0.326 | | 2.029(1.253-3.286) | 0.004^⁎^ | | 2.026(1.248-3.289) | 0.004^⁎^ |
|  | Overdominant | 0.969(0.740-1.270) | 0.820 | | 0.954(0.727-1.251) | 0.732 | | 0.737(0.509-1.067) | 0.106 | | 0.746(0.515-1.080) | 0.121 | | 1.096(0.831-1.446) | 0.517 | | 1.081(0.818-1.427) | 0.584 |
|  | Addictive | 1.253(1.026-1.529) | 0.027^⁎^ | | 1.262(1.033-1.542) | 0.023^⁎^ | | 1.395(0.993-1.960) | 0.055 | | 1.379(0.980-1.939) | 0.065 | | 1.228(0.994-1.516) | 0.057 | | 1.237(1.001-1.528) | 0.049^⁎^ |
|  | Allele | 1.255(1.028-1.532) | 0.026^⁎^ | |  |  | | 1.395(0.994-1.958) | 0.054 | |  |  | | 1.230(0.995-1.519) | 0.055 | |  |  |
| Sex |  |  |  |  |  |  |  |  |  |  |  |  |  |  |  |  |  |  |
| Male | Codominant | 0.954(0.688-1.321) | 0.775 | | 0.961(0.692-1.334) | 0.811 | | 1.414(0.935-2.140) | 0.101 | | 1.435(0.945-2.177) | 0.090 | | 0.905(0.654-1.253) | 0.547 | | 0.957(0.683-1.342) | 0.799 |
|  |  | 1.283(0.778-2.116) | 0.329 | | 1.304(0.787-2.162) | 0.303 | | 0.881(0.181-4.276) | 0.875 | | 0.950(0.192-4.704) | 0.950 | | 1.700(0.945-3.058) | 0.077 | | 1.841(1.036-3.269) | 0.037^⁎^ |
|  | Dominant | 1.012(0.743-1.378) | 0.940 | | 1.020(0.748-1.393) | 0.899 | | 1.373(0.917-2.057) | 0.124 | | 1.400(0.931-2.104) | 0.106 | | 0.998(0.734-1.357) | 0.991 | | 1.072(0.780-1.473) | 0.669 |
|  | Recessive | 0.761(0.474-1.219) | 0.256 | | 1.331(0.827-2.145) | 0.239 | | 0.835(0.172-4.050) | 0.823 | | 0.898(0.182-4.439) | 0.895 | | 1.772(1.000-3.140) | 0.050^⁎^ | | 1.875(1.075-3.271) | 0.027^⁎^ |
|  | Overdominant | 1.104(0.812-1.502) | 0.528 | | 1.100(0.807-1.498) | 0.547 | | 0.706(0.467-1.068) | 0.099 | | 0.697(0.459-1.057) | 0.089 | | 1.175(0.856-1.612) | 0.319 | | 1.133(0.817-1.570) | 0.455 |
|  | Addictive | 1.072(0.851-1.351) | 0.555 | | 1.080(0.856-1.364) | 0.516 | | 1.284(0.891-1.852) | 0.180 | | 1.313(0.907-1.901) | 0.150 | | 1.103(0.861-1.412) | 0.437 | | 1.174(0.915-1.507) | 0.207 |
|  | Allele | 1.070(0.852-1.343) | 0.561 | |  |  | | 1.301(0.894-1.893) | 0.168 | |  |  | | 1.100(0.862-1.405) | 0.443 | |  |  |
| Female | Codominant | 1.006(0.723-1.401) | 0.970 | | 1.079(0.769-1.515) | 0.659 | | 1.330(0.867-2.041) | 0.191 | | 1.344(0.866-2.084) | 0.187 | | 0.934(0.672-1.298) | 0.684 | | 0.923(0.665-1.281) | 0.632 |
|  |  | 1.353(0.826-2.215) | 0.230 | | 1.568(0.941-2.613) | 0.084 | | 2.423(0.537-10.927) | 0.250 | | 2.015(0.433-9.367) | 0.372 | | 1.673(0.955-2.929) | 0.072 | | 1.683(0.929-3.047) | 0.086 |
|  | Dominant | 1.072(0.785-1.463) | 0.661 | | 1.167(0.848-1.606) | 0.343 | | 1.379(0.910-2.089) | 0.129 | | 1.378(0.900-2.110) | 0.140 | | 1.032(0.757-1.407) | 0.841 | | 1.013(0.743-1.382) | 0.935 |
|  | Recessive | 1.349(0.847-2.147) | 0.208 | | 1.510(0.933-2.446) | 0.094 | | 2.324(0.516-10.466) | 0.272 | | 1.929(0.415-8.953) | 0.402 | | 1.722(1.001-2.964) | 0.050^⁎^ | | 1.740(0.976-3.102) | 0.060 |
|  | Overdominant | 1.059(0.776-1.447) | 0.717 | | 1.016(0.738-1.398) | 0.924 | | 0.759(0.495-1.164) | 0.207 | | 0.750(0.484-1.163) | 0.199 | | 1.147(0.834-1.577) | 0.398 | | 1.152(0.837-1.584) | 0.386 |
|  | Addictive | 1.114(0.886-1.401) | 0.357 | | 1.197(0.945-1.515) | 0.136 | | 1.379(0.944-2.015) | 0.097 | | 1.361(0.923-2.007) | 0.120 | | 1.130(0.885-1.441) | 0.327 | | 1.110(0.865-1.425) | 0.411 |
|  | Allele | 1.115(0.886-1.403) | 0.355 | |  |  | | 1.392(0.948-2.046) | 0.090 | |  |  | | 1.128(0.885-1.438) | 0.329 | |  |  |
